# Supplementary material for: Stabilization of Palygorskite Aqueous Suspensions Using Bio-Based and Synthetic Polyelectrolytes
Source: Polymers (Basel). 2020 Dec 30;13(1):129. doi: 10.3390/polym13010129 (PMC7795911; doi:10.3390/polym13010129)
Supplement: Supplementary file 1 [file polymers-13-00129-s001.pdf]

Article

Supplementary Materials

Stabilization of Palygorskite Aqueous Suspensions Using Bio-Based and Synthetic Polyelectrolytes

Eduardo Ferraz [1,2], Luís Alves [3,*], Pedro Sanguino [4], Julio Santarén [5], Maria G. Rasteiro [3] and José A. F. Gamelas [3,*]

[1] Techn&Art, Polytechnic Institute of Tomar, Quinta do Contador, Estrada da Serra, PT - 2300-313 Tomar, Portugal; ejmoferraz@ipt.pt
[2] Geobiotec, University of Aveiro, Geosciences Department, Campus Universitário de Santiago, PT - 3810-193 Aveiro, Portugal
[3] CIEPQPF, Department of Chemical Engineering, University of Coimbra, Rua Sílvio Lima, Pólo II, PT - 3030-790 Coimbra, Portugal; mgr@eq.uc.pt
[4] CEMMPRE, Mechanical Engineering Department, University of Coimbra, Rua Luís Reis Santos, Pólo II, PT - 3030-788 Coimbra, Portugal; pesang@sapo.pt
[5] TOLSA, SA, Research & Technology for New Businesses, Ctra. de Madrid a Rivas Jarama, 35, ES - 28031 Madrid, Spain; jsantaren@tolsa.com
* Correspondence: luisalves@ci.uc.pt (L.A.); jafgas@eq.uc.pt (J.A.F.G.)

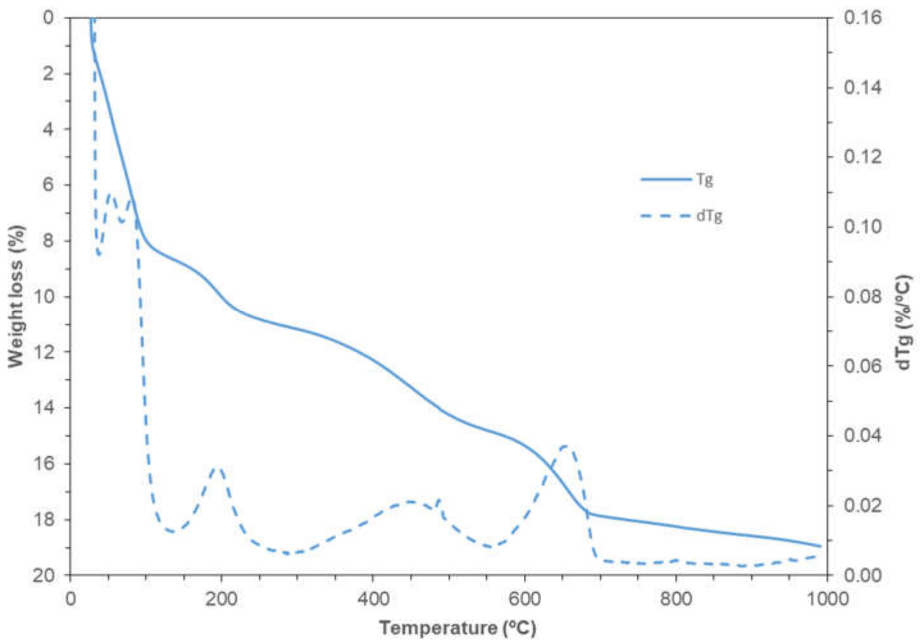

Figure S1. Thermogravimetry (solid) and derivative (dashed) curves for the palygorskite.

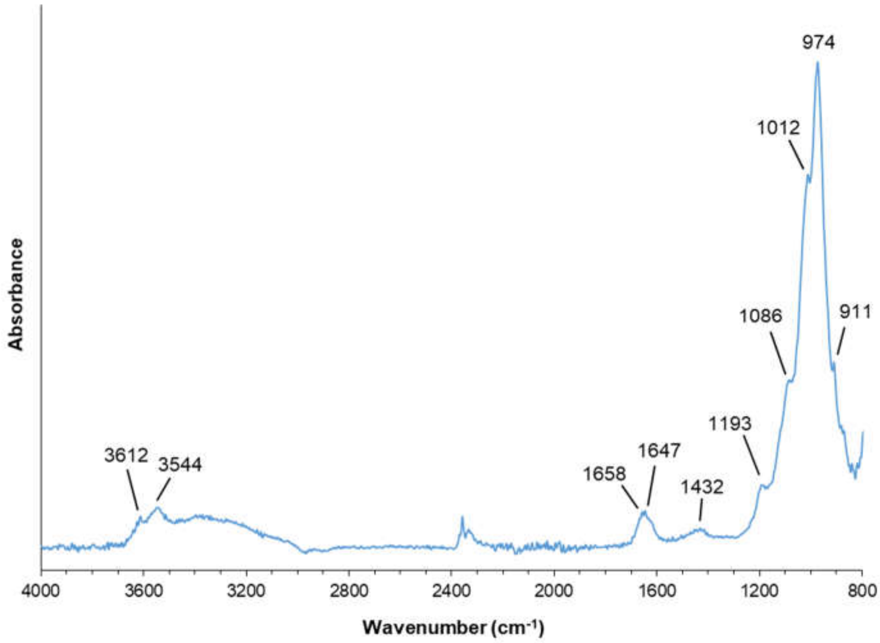

Figure S2. FTIR spectrum of the palygorskite.

a)

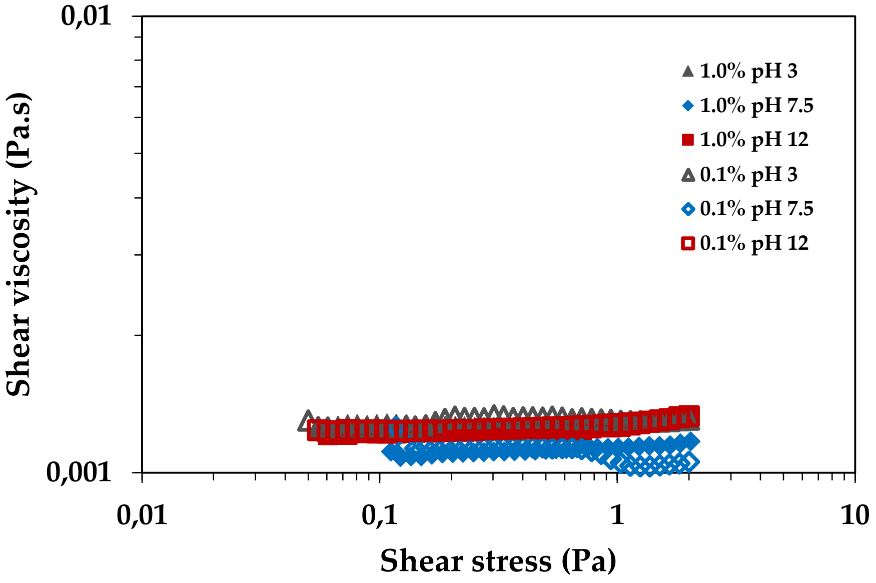

b)

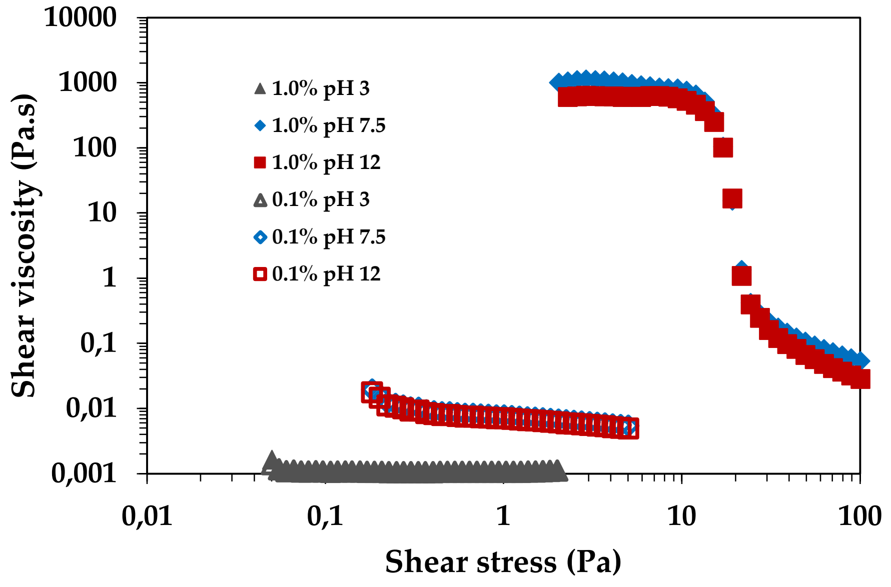

Shear viscosity (Pa.s)
10000
1000
100
10
1
0,1
0,01
0,001
0,01
0,1
1
10
100
Shear stress (Pa)
1.0% pH 3
1.0% pH 7.5
1.0% pH 12
0.1% pH 3
0.1% pH 7.5
0.1% pH 12

c)

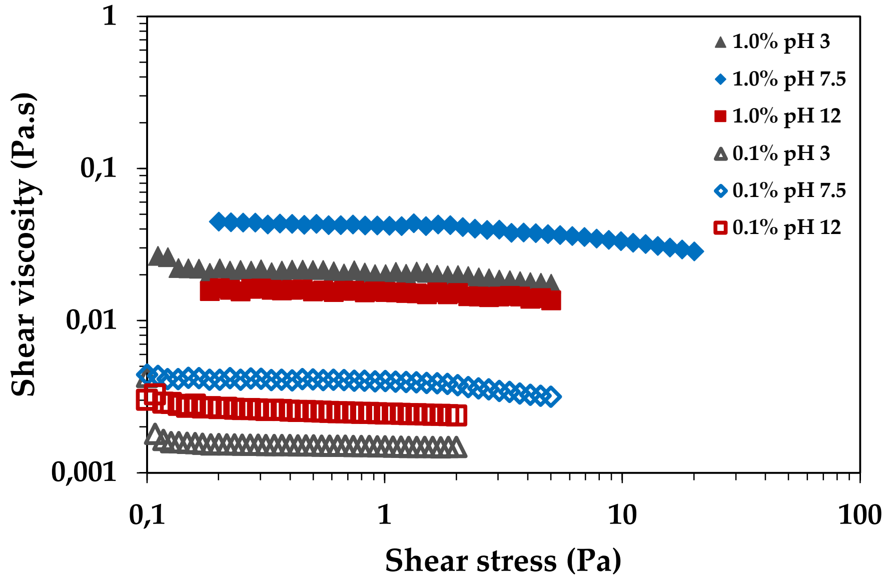

Shear viscosity (Pa.s)
1
0,1
0,01
0,001
0,1
1
10
100
Shear stress (Pa)
1.0% pH 3
1.0% pH 7.5
1.0% pH 12
0.1% pH 3
0.1% pH 7.5
0.1% pH 12

d)

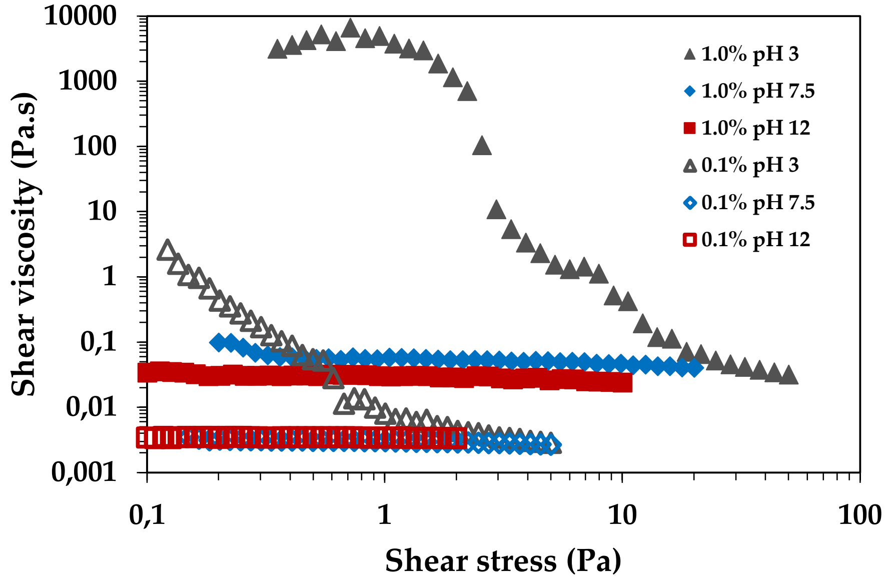

Shear viscosity (Pa.s)
10000
1000
100
10
1
0,1
0,01
0,001
0,1
1
10
100
Shear stress (Pa)
1.0% pH 3
1.0% pH 7.5
1.0% pH 12
0.1% pH 3
0.1% pH 7.5
0.1% pH 12

Figure S3. Flow curves of the four different dispersing agents at two different concentrations (1.0 wt% and 0.1 wt%), at 20ºC. a) Polyphosphate; b) Polyacrylate; c) CMC; d) Alginate.

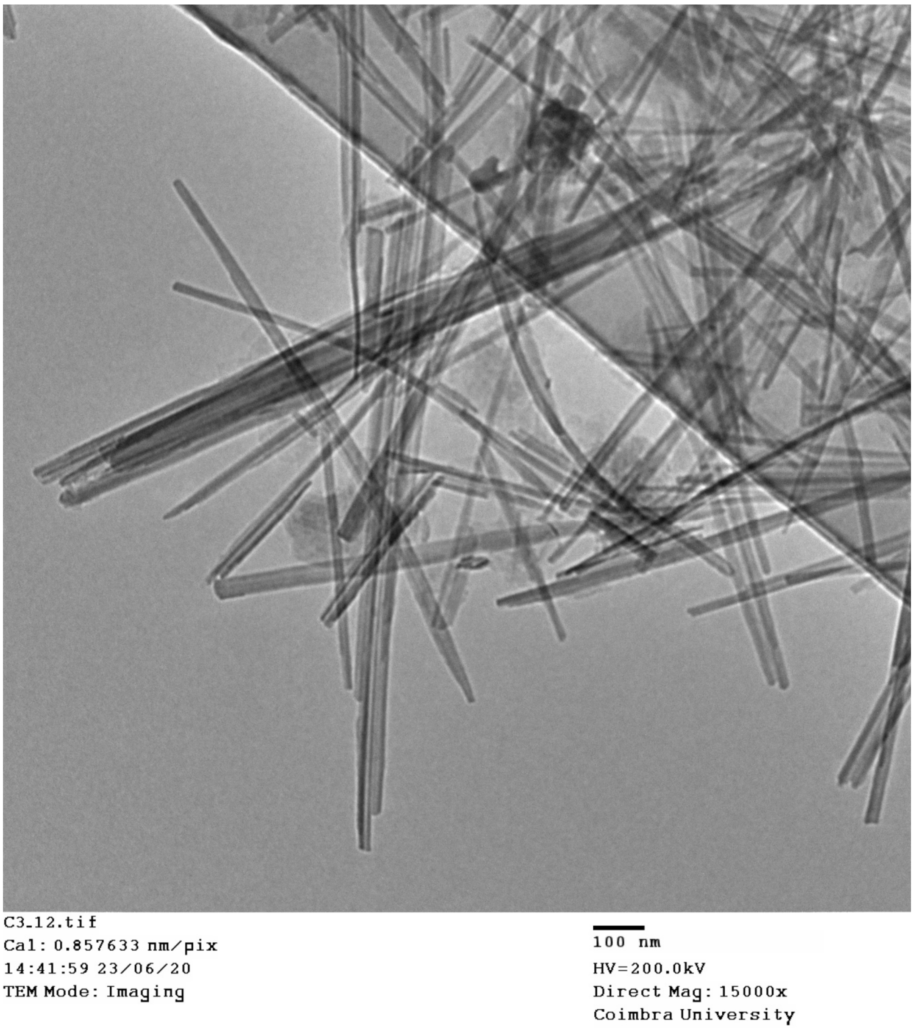

Figure S4. TEM image of 0.05 wt% palygorskite suspension prepared using the ultrasonic probe at pH ca. 8, with polyacrylate, taken 20 days after preparation of the suspension.
